# Mechanisms of aerobic exercise effects on the gut microbiota and its metabolites in anxiety disorders

**DOI:** 10.3389/fmicb.2025.1721497

**Published:** 2025-12-11

**Authors:** Yalin Zheng, Yaqi Qu, Mingchen Yao, Kaixuan Li, Yani Dong, Xinru Xing, Tingwu Yang, Hao Guo, Peng Huang

**Affiliations:** School of Sports Medicine and Rehabilitation, Beijing Sport University, Beijing, China

**Keywords:** anxiety disorders, aerobic exercise, gut microbiota, metabolites, microbiota-gut-brain axis

## Abstract

Anxiety disorders are prevalent and disabling conditions that frequently co-occur with major depression, alcohol and substance abuse disorders, and personality disorders. While psychopharmacological treatments and cognitive-behavioral therapy are the primary interventions for anxiety disorders, up to 30% of patients exhibit inadequate treatment responses, and approximately 40% relapse within the first year after discontinuing medication. Moreover, medication-related side effects have a significant impact on patients’ quality of life. These limitations highlight the urgent need for more comprehensive research into the underlying pathophysiology of anxiety disorders to facilitate the development of more effective therapeutic strategies. In this context, aerobic exercise has consistently been linked to improvements in physical health, life satisfaction, cognitive functioning, and psychological wellbeing. Aerobic exercise may serve as an effective and cost-efficient alternative for managing various anxiety disorders. This review explores the potential mechanisms through which aerobic exercise influences anxiety disorders, focusing on its effects on gut microbiota and related metabolites. The findings aim to propose novel therapeutic strategies for patients with anxiety disorders and provide a theoretical foundation for future research in this field.

## 1 Introduction

Anxiety disorders are common and debilitating conditions that frequently co-occur with major depression, alcohol and substance abuse disorders, and personality disorders. With rapid socioeconomic development and an aging population, individuals face mounting life pressures and increasing healthcare demands, leading to a steady rise in anxiety disorders (Penninx et al., 2021). According to the World Health Organization (WHO), anxiety disorders affect about 970 million people with mental illnesses globally, and the burden of disease caused by anxiety disorders ranks second among all types of mental disorders (Freeman, 2022; World Mental Health Today: Latest Data, 2025). Moreover, individuals with anxiety disorders are at a significantly elevated risk of suicide, with an estimated suicide rate of 20% (Bachmann, 2018; De La Vega et al., 2018). Thus, anxiety disorders represent a critical challenge to global physical and mental health.

Psychotropic medications are first-line treatments for anxiety disorders (Zwanzger et al., 2021). Antidepressants, particularly Selective Serotonin Reuptake Inhibitors (SSRIs) and Serotonin-Norepinephrine Reuptake Inhibitors (SNRIs), are central in pharmacotherapy. Their efficacy is well-established through numerous studies and meta-analyses, supporting their use as first-choice treatments (Bandelow, 2020). However, approximately 30% of patients show inadequate response to these medications (Zwanzger et al., 2021), and nearly 40% relapse within one year after discontinuation (Ravindran and Stein, 2010). Common side effects of SSRIs include orgasmic dysfunction and delayed ejaculation (Montejo et al., 2015; Rothmore, 2020). Long-term antidepressant use has also been associated with increased risks of atherosclerotic cardiovascular disease and seizures (Kim et al., 2024). In addition to pharmacological interventions, cognitive-behavioral therapy (CBT), grounded in neuroscientific, experimental, and psychological research, is considered the psychotherapy of choice for most individuals with anxiety disorders. Extensive research has consistently demonstrated its efficacy and effectiveness in treating these conditions (Borza, 2017; Kodal et al., 2018). Nonetheless, its implementation is hampered by therapist shortages, long waiting times, and high costs, which limit accessibility (Baumann et al., 2020). Untreated anxiety disorders often follow a chronic, relapsing course. Existing treatments are effective in fewer than half of patients, with outcomes varying widely (Simpson et al., 2021). Given these limitations, there is an urgent need to deepen our understanding of the underlying pathophysiology of anxiety disorders to facilitate the development of more effective therapeutic options.

The pathogenesis of anxiety disorders remains incompletely understood. However, recent years have accelerated research on the gut microbiota, with growing evidence highlighting its influence on host behavior and brain function. The gut microbiota has emerged as a crucial determinant of human health and disease, vital in maintaining host homeostasis. Notably, the microbiota-gut-brain (MGB) axis has been implicated in the pathogenesis of anxiety disorders through neural, metabolic, hormonal, and immune-mediated pathways. Evidence from fecal microbiota transplantation (FMT) studies indicates that gut microbiota can transfer various behavioral phenotypes, including anxiety-like behaviors, underscoring its role in anxiety regulation (Li et al., 2019). Moreover, the gut microbiota modulates the production of neurotransmitters and their precursors (Duranti et al., 2020), as well as the expression of brain-derived neurotrophic factor (BDNF) in the brainstem and hypothalamus, thereby influencing central nervous system (CNS) plasticity (Clarke et al., 2013; Schéle et al., 2013). Gut microbiota metabolites, particularly short-chain fatty acids (SCFAs), contribute to host health through multiple mechanisms. They activate vagus nerve (VN)-mediated CNS signaling via enteroendocrine pathways while enhancing intestinal barrier function through mucus production and exerting systemic anti-inflammatory effects (Han et al., 2023; Zhang et al., 2023b). Collectively, these findings underscore the critical role of the gut microbiota in the development and progression of anxiety disorders. The modulatory effects of mental disorders through modulation of the gut macrogenome have been extensively studied, and recently, researchers have focused on the mechanisms by which exercise modulates anxiety through modulation of the MGB axis.

Exercise has consistently been associated with improvements in physical health, life satisfaction, cognitive function, and mental health, making it a practical and cost-efficient alternative for treating various anxiety disorders (Carek et al., 2011). Current research suggests that the positive effects of aerobic exercise are primarily related to enhancements in colonic health, increased microbiota diversity, and the balance between beneficial and pathogenic bacterial communities (Dalton et al., 2019). Mitchell et al. (2019) found that exercise positively impacts gut microbiota by increasing butyrate-producing bacteria. These findings underscore the critical role of aerobic exercise in regulating the MGB axis.

However, the potential mechanisms underlying the anti-anxiety effects of aerobic exercise remain unclear and require further investigation. This study aims to explore the neurobiological mechanisms by which aerobic exercise influences anxiety disorders, attempt to elucidate potential therapeutic pathways, provide new strategies for future treatment, and lay a theoretical foundation for subsequent related research.

## 2 Methods

A comprehensive literature search was conducted to identify relevant studies published up to June 2025. The electronic databases utilized included PubMed, Web of Science, and Scopus. The search strategy was designed to encompass three core conceptual domains: (1) anxiety disorders, (2) gut microbiota and their metabolites, and (3) aerobic exercise. The following key search terms and their Boolean operators were employed: (“anxiety disorder” OR “anxiety” OR “anxious”) AND (“gut microb” OR “gut microbiome” OR “gut microbiota” OR “microbiome-gut-brain axis” OR “short-chain fatty acid*” OR SCFAs) AND (“aerobic exercise” OR “physical activity” OR “running” OR “treadmill”).

The retrieved records were initially screened based on the titles and abstracts of the literature, followed by full-text evaluation of potentially eligible articles. Inclusion criteria were (1) original research papers or reviews (including systematic reviews and meta-analyses) published in peer-reviewed journals; (2) studies involving associations between aerobic exercise, gut microbiota/metabolites, and anxiety or anxiety-like behaviors; and (3) studies in human subjects or animal models. Exclusion criteria were (1) studies published in languages other than English; (2) conference abstracts, editorials, or book chapters; and (3) studies focusing only on exercise or microbiota without an explicit association with anxiety.

## 3 Relationship between the gut microbiota and its metabolites and anxiety disorders

The gut microbiota, consisting of bacteria, bacteriophages, viruses, fungi, protozoa, and archaea, plays a critical role in human health and disease (Yatsunenko et al., 2012). Estimates suggest that the number of bacteria inhabiting the gastrointestinal tract of healthy humans ranges from 10^13^ to 10^14^ microorganisms (Fu et al., 2022). They were mainly classified into 11 phyla, of which Proteobacteria, Firmicutes, Actinobacteria, and Bacteroidetes comprised over 90% of the microbiome, whereas Fusobacteria and Verrucomicrobia phyla were of low abundance (Bilen et al., 2018; Hugon et al., 2015). Numerous studies have underscored the pivotal role of the gut microbiota in developing the host’s adaptive immune system and its influence on neuroplasticity in the CNS (Deng et al., 2021a; Zou et al., 2023). Increasing research on the gut microbiome is uncovering its crucial role in regulating brain function and mental health (Feng et al., 2023; Shan et al., 2020; Wang et al., 2022a).

### 3.1 Association between gut microbiota composition and anxiety disorders

Anxiety is a highly prevalent comorbid mental health condition that is strongly associated with reduced gut microbiota diversity and abundance. Individuals with anxiety disorders typically exhibit lower microbiota diversity and abundance compared to healthy individuals (Chen et al., 2019b).

At the phylum level, Firmicutes and Bacteroidetes are the most significantly affected by anxiety disorders. Decreased levels of Firmicutes and increased levels of Bacteroidetes have been consistently observed in large-scale cohort studies of individuals with anxiety disorders, as well as in animal studies (Chen et al., 2019a; Deng et al., 2021a). In an animal study, Li et al. (2019) showed that transplantation of the fecal microbiota of donor mice from a CUMS model into healthy C57BL/6 recipient mice was sufficient to transmit anxiety-like and depression-like behaviors, and that dysbiosis of the gut microbiota was manifested by an increase in the relative abundance of Sutterellaceae, Ruminococcaceae, and Desulfovivionaceae. Notably, the increases in Bifidobacteriaceae and Lactobacillaceae were particularly dramatic. However, in complete contrast to the findings of Deng et al. (2021), who showed a decrease in the relative abundance of Sutterellaceae, Ruminococcaceae, and Desulfovibrionaceae in the domestic layer and a significant increase in the abundance of Lactobacillaceae. Taking into account the differences that may be caused by differences in how anxiety-like mice are induced, the changes in gut microbiota were not the same. However, they all showed the same anxiety-like behaviors in behavioral tests. This suggests that future research should focus on optimizing the induction of animal models, including both behavioral and microbiota features, that more accurately reflect the real-world conditions of patients with anxiety disorders. Such models could help clarify the pathways through which the gut microbiota influences anxiety disorders and contribute to the development of more effective medications with fewer side effects.

### 3.2 Association between gut microbiota metabolites and anxiety disorders

Gut microbiota produce a wide variety of metabolites capable of influencing host physiology and brain function, including SCFAs, neurotransmitters [e.g., gamma-aminobutyric acid (GABA), serotonin (5-HT)], tryptophan derivatives, and bile acids (Dalal et al., 2025). Although this network of metabolites collectively shapes the host’s mental state, SCFAs - particularly acetic, propionic, and butyric acids - have received a great deal of attention in the field of anxiety disorders research (Lobzhanidze et al., 2019a; Needham et al., 2022; Wang et al., 2012). This has been attributed to their wide-ranging effects on anxiety-related immune, endocrine, and neural pathways via the MGB axis. Therefore, this subsection focuses on the contribution of SCFAs in the regulation of anxiety disorders.

SCFAs, including butyrate, propionate, and acetate, are produced in the colon through bacterial fermentation of dietary fiber and serve as a primary energy source for bacteria in the digestive system (Tan et al., 2014). As key microbiota-gut-brain messengers, SCFAs play a crucial role in mental health. The observation of reduced SCFAs levels in animal models of anxiety highlights the significant involvement of the gut microbiota in anxiety disorders (Lobzhanidze et al., 2019b; Zhang et al., 2023b).

Among SCFAs, butyrate has been the most extensively studied. The fermentation of resistant starch is thought to significantly contribute to butyrate production in the colon, primarily originating from *Ruminococcus bromii*, as its absence substantially reduces resistant starch fermentation (Ze et al., 2012). Butyrate is present at high concentrations in the intestinal lumen, serving as a significant energy source for colonocytes, and is the most potent SCFAs in inhibiting histone deacetylase (HDAC) (Dalile et al., 2024). Studies have shown that butyrate prevents colorectal cancer and inflammation by inhibiting HDAC (Flint et al., 2012). Additionally, butyrate crosses the Blood–Brain Barrier (BBB) and inhibits HDAC1 and HDAC2, enzymes regulating the stress response (Peng et al., 2021; Sakharkar et al., 2014). Intracerebral inhibition of HDAC in regions such as the prefrontal cortex, nucleus ambiguus, hippocampus, and amygdala reverses stress-induced anxiety-like behavior in rodents (Lu et al., 2023). Most gut microbiota can produce acetate, including *Akkermansia muciniphila*, *Bacteroides*, and *Bifidobacteria* (Koh et al., 2016). Propionate is primarily produced by a few dominant genera, including *Akkermansia muciniphila*. *Bacteroides vulgatus* and *Bacteroides thetaiotaomicron* also produce propionate through the succinate pathway (Koh et al., 2016; Morrison and Preston, 2016). Acetic acid crosses the BBB, inhibits HDAC in the prefrontal cortex, nucleus ambiguus, hippocampus, and amygdala, suppresses microglia activation, and reverses stress-induced anxiety-like behavior in rodents (Guan et al., 2022; Inamoto et al., 2023; Lu et al., 2023). In addition, propionic acid was able to induce a decrease in the number of neurons in the amygdala and an increase in the activation of astrocytes and microglia (Lobzhanidze et al., 2019a). However, in a study in stressed mice, propionic acid levels were found to be significantly reduced (Wah et al., 2019), suggesting that the underlying mechanisms of anxiety from acute stress and chronic unpredictable stress may not be identical and remain to be further explored.

As detailed in Section 2.2, SCFAs, particularly butyrate, are known to cross the BBB and play a crucial role in maintaining its integrity. The integrity of the BBB is critical for preventing the entry of pro-inflammatory cytokines and other neurotoxic substances from the periphery into the brain. A ‘leaky’ BBB is implicated in neuroinflammation, which is a key pathophysiological mechanism in anxiety disorders. Moreover, butyrate has been shown to improve anxiety-like behaviors caused by chronic stress. In addition, SCFAs can modulate microglia activation, influencing the release of pro-inflammatory factors, such as IL-1β, IL-6, and TNF-*α*, which can further impact neuroinflammation and anxiety-like behaviors (Caetano-Silva et al., 2023; Wei et al., 2023).

However, inconsistencies remain in the field, such as conflicting variations in the Firmicutes/Bacteroidetes ratio reported in different studies, which may be attributed to animal models, research methods, and individual differences. In Summary, this evidence indicates that SCFAs modulate BBB permeability, neurogenesis, and behavioral responses. Nevertheless, the microbial mechanisms underlying stress-induced anxiety and SCFAs’ therapeutic potential remain poorly understood. Well-controlled longitudinal human studies in healthy and clinical populations, combined with translational preclinical models, are urgently needed to elucidate these mechanisms and evaluate SCFAs’ therapeutic efficacy across developmental stages and administration routes.

## 4 The influence of gut microbiota and its metabolites on anxiety through the microbiota-gut-brain axis

The pathogenesis of anxiety disorders involves complex, multifactorial mechanisms. Emerging evidence highlights the MGB axis as a pivotal etiological component (Deng et al., 2021b). Preclinical studies consistently show that microbial dysbiosis potently modulates anxiety-like and stress-related behaviors, implicating the MGB axis in anxiety disorder vulnerability. Although the exact mechanisms remain elusive, current research suggests microbiota may regulate brain function through the gut-brain axis by simultaneously influencing neural, immune, and endocrine pathways (Malan-Muller et al., 2018; Su et al., 2022).

### 4.1 The influence of gut microbiota and its metabolites on the nervous system

**Figure 1:**
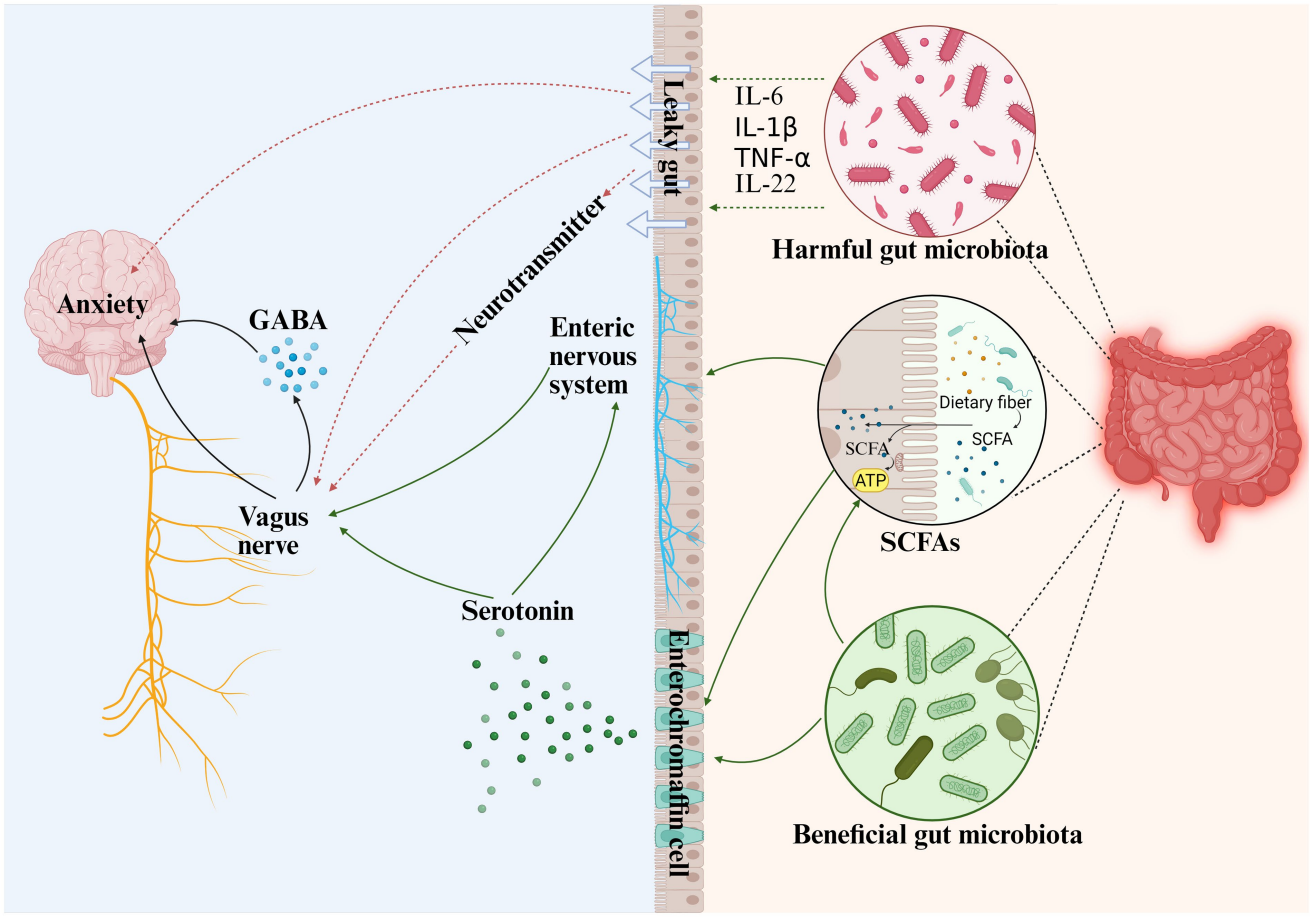
Gut microbiota regulates anxiety through the neurological system of the gut-brain axis. (1) Inflammatory factors secreted by harmful intestinal microbiota damage the intestinal barrier and cause “leaky gut,” and the inflammatory factors directly activate the vagus nerve. (2) Stimulation of enteroendocrine cells and the release of neuroactive mediators also indirectly activate the vagus nerve, which promotes the secretion of inhibitory neurotransmitter GABA in the brain and thus affects the central nervous system. (3) SCFAs, a metabolite of the intestinal microbiota, affect the synthesis and secretion of serotonin from enterochromaffin cells, which in turn affects the VNS. (4) The synthesis and secretion of serotonin stimulates enteric neurogenesis, increases the number of enteric glial cells and neurons, and promotes colon motility, which, through the ENS, affects the VNS, which in turn affects the onset of anxiety. (Green lines are facilitative; red lines are inhibitory). GABA, gamma-aminobutyric acid; SCFAs, short-chain fatty acids; IL-6, Interleukin-6, IL-1β, Interleukin-1β; IL-22, Interleukin-22; TNF-α, tumor necrosis factor-α; IFN-*γ*, Interferon-γ (Created with BioRender.com).

Neuromodulation occurs primarily through impulse transmission within the autonomic nervous system (ANS), including the vagus nerve and the enteric nervous system (ENS) (Figure 1).

The VN, the body’s longest cranial nerve, regulates intestinal physiology and modulates cardiovascular, respiratory, immune, and endocrine functions (Browning et al., 2017). Recently, its role in mood regulation, including anxiety disorders, has attracted growing interest. Referred to as the “great wandering protector,” the VN is essential for maintaining organismal homeostasis and is increasingly studied as a therapeutic target for various diseases (Bonaz et al., 2018).

VN dysfunction leads to abnormal neurogenesis, stress responses, and anxiety. The gut microbiota can activate the vagus nerve directly when the intestinal barrier is compromised and indirectly after stimulation and release of neuroactive mediators in enteroendocrine cells or gut-associated lymphoid tissue (Browning et al., 2017). For instance, inducing enteric infection in mice using *Citrobacter rodentium* increased anxiety-like behavior in a vagal-dependent manner (Lyte et al., 2006). Conversely, in mice with infectious colitis, *Bifidobacterium longum* NCC3001 ameliorated anxiety-like behavior in infectious colitis models by activating vagal pathways to the central nervous system (Bercik et al., 2011). More recently, Zou et al. (2024) demonstrated that the unilateral vagotomy reduced *S. typhimurium*-induced anxiety-like behaviors in mice, lowered serum endotoxin, and decreased inflammatory cytokines (IL-6, IL-1β, TNF-*α*, IL-22, CXCL1) in the gut and brain. Together, these findings underscore that vagal integrity is indispensable for mediating gut microbiota-induced anxiety-like behaviors.

Fluoxetine, a frequently prescribed medication for anxiety, has been shown to modulate anxiety symptoms via the vagus nerve and can result in a decreased Firmicutes/Bacteroidetes ratio (Joo and Kim, 2023). Additionally, vagus nerve stimulation (VNS) has been found to offer therapeutic benefits for anxiety disorders (Yue et al., 2023). A study based on a rat model showed that delivering 5 sets of VNS training sessions produced a stronger anxiolytic effect than delivering a single set of VNS (Mathew et al., 2020). However, the mechanisms underlying these treatments are not yet fully understood, and their relationship with the MGB axis remains a topic of debate (Breit et al., 2018). Future research should employ more rigorous experimental designs to evaluate the safety and potential efficacy of VNS in patients with anxiety disorders, investigate the underlying mechanisms, and conduct in-depth comparisons of VNS stimulation parameters and dose effects. Such studies are essential to optimizing the therapeutic benefits of VNS for patients with anxiety disorders.

The ENS arises from neural crest cells of primarily vagal origin and consists of a nerve plexus embedded in the intestinal wall, extending across the whole gastrointestinal tract from the esophagus to the anus. The ENS produces over 30 neurotransmitters and contains more neurons than the spinal cord. Often referred to as the “second brain,” the ENS shares structural, functional, and chemical similarities with the brain (Schemann and Neunlist, 2004). ENS dysfunction is associated with gastrointestinal symptoms—such as severe constipation, anorexia, and delayed gastric emptying—commonly comorbid with anxiety disorders (Chalazonitis and Rao, 2018). These associations have prompted exploration of intestinal myoelectrical activity as a potential diagnostic marker for anxiety (Li et al., 2023). Altered mesenteric nerve firing has been observed in GF mice compared to specific-pathogen-free (SPF) and recolonized controls (McVey Neufeld et al., 2015). Vicentini et al. (2021) reported that antibiotic-induced microbiota depletion led to structural and functional gut impairments, including increased permeability, loss of enteric neurons and glia in the ileum and proximal colon. Microbiota restoration reversed these deficits and stimulated enteric neurogenesis. Supplementation with SCFAs, particularly butyrate, also restored neuronal numbers and promoted neuroplasticity, increasing cholinergic myenteric neurons and enhancing colonic motility in adult rats (Suply et al., 2012).

The above findings suggest that gut microbes and their metabolites play an important role in ENS plasticity and are essential for maintaining the integrity of the ENS. Future research should expand ENS sampling to study microbial-driven regulation of intestinal permeability and identify key SCFAs for treating anxiety disorders.

### 4.2 The influence of gut microbiota and its metabolites on the endocrine system

#### 4.2.1 Hypothalamic–pituitary–adrenal (HPA) axis

**Figure 2:**
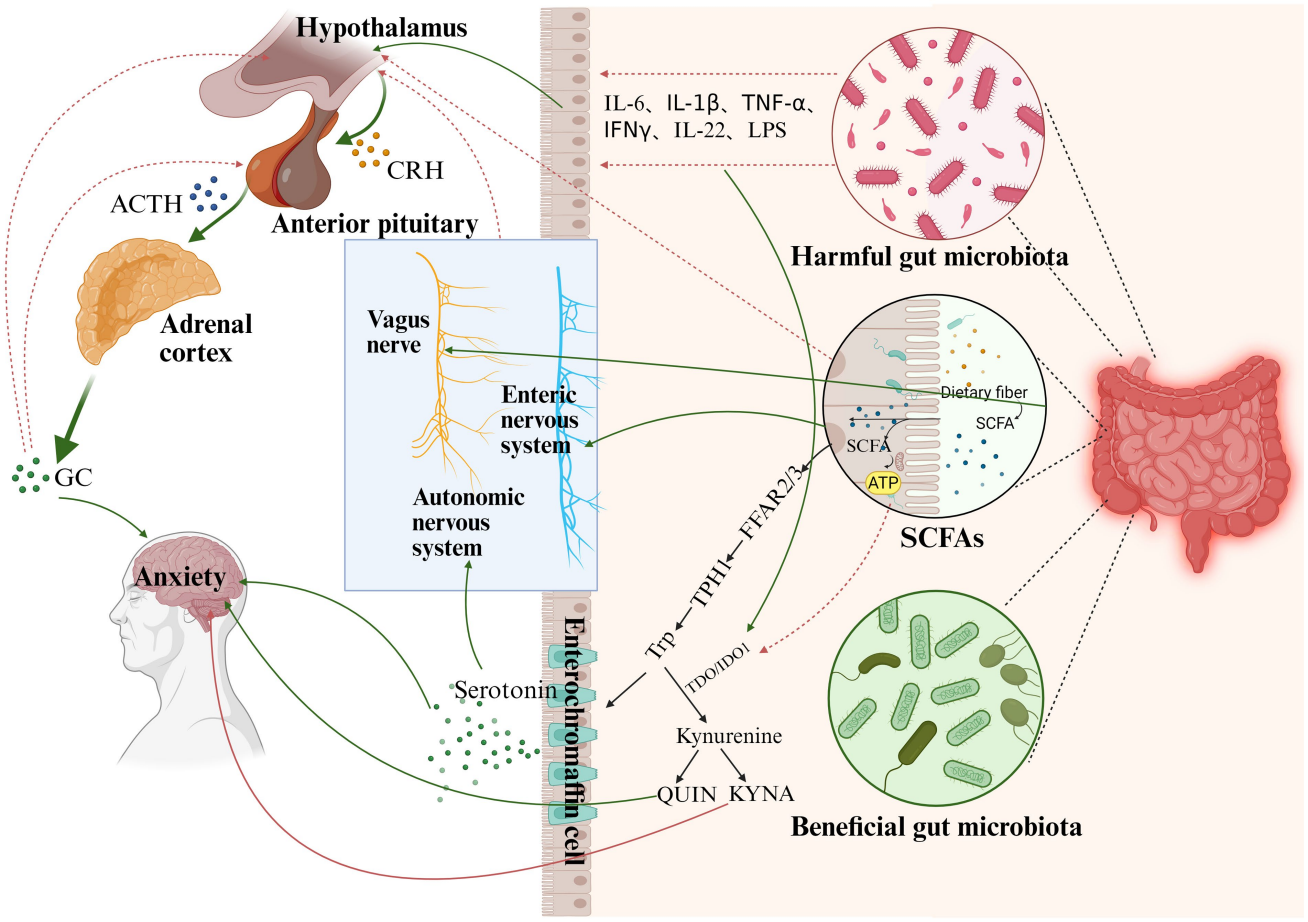
The gut microbiota may regulate the endocrine system through the following pathways. (1) Inflammatory factors and lipopolysaccharides produced by the gut microbiota promote the production of CRH in the hypothalamus and regulate HPA axis function. (2) SCFAs, a metabolite of the gut microbiota, promote the transcription of TPH1 in the colon, affect tryptophan levels, and influence serotonin production by enterochromaffin cells. Serotonin acts on the ANS, thereby altering the activation state of the HPA axis. At the same time, SCFAs cross the blood–brain barrier and inhibit the activation of the HPA axis (3). Serotonin also directly affects neurogenesis and neuroplasticity in the central nervous system, with effects on depression and anxiety. (4) An increase in harmful gut microbiota promotes the release of pro-inflammatory cytokines (e.g., IL-1, IL-6, and TNF-α), which in turn act on the hypothalamus and anterior pituitary, leading to aberrant activation of the HPA axis, which has effects on neurogenesis and neuroplasticity, and ultimately on anxiety. It also promotes the expression of TDO/IDO1 in tryptophan metabolism, which promotes the activation of the kynurenine pathway and affects anxiety. (5) SCFAs inhibit the expression of TDO/IDO1 in tryptophan metabolism, which inhibits the activation of the kynurenine pathway, promotes the transfer of tryptophan metabolism to the serotonin pathway, and alleviates the occurrence of anxiety (green lines are facilitative, red lines are inhibitory). CRH, corticotropin-releasing hormone; ACTH, adrenocorticotropic hormone; GC, glucocorticoid; SCFAs, short-chain fatty acids; Trp, tryptophan; TPH1, tryptophan hydroxylase 1; FFAR2,3, free fatty acid receptors2,3; TDO, tryptophan-2,3-dioxygenase; IDO1, indoleamine-2,3-dioxygenase; QUIN, quinolinic acid; KYNA, quinaldic acid; IL-6, interleukin-6, IL-1β, interleukin-1β; IL-22, interleukin-22; TNF-α, tumor necrosis factor-α; IFN-γ, interferon-γ (Created with BioRender.com).

Research conducted over the past few decades has demonstrated that neurodevelopmental and mood disorders are closely linked to disruptions in the balance of the endocrine system. Notably, anxiety is often an early manifestation or a characteristic symptom of several endocrine disorders (Goncerz and Wójcik, 2025; Hall and Hall, 1999) (Figure 2). Endocrine dysfunction frequently induces cognitive and behavioral alterations, particularly anxiety and cognitive deficits. Elucidating gut microbiota-metabolite-endocrine interactions may uncover fundamental mechanisms underlying anxiety disorder pathogenesis (Saeidi et al., 2025).

Indeed, the ability to adapt to and respond to environmental stimuli is a fundamental function for all organisms. The HPA axis plays a central role in the stress response and represents the neuroendocrine cascade that leads to the synthesis and secretion of glucocorticoids. Upon encountering a stressful event, the HPA axis is activated, triggering the hypothalamus’s paraventricular nucleus (PVN) to release corticotropin-releasing hormone (CRH). This, in turn, signals the anterior pituitary gland to secrete adrenocorticotropic hormone (ACTH) into the bloodstream. ACTH then reaches the adrenal glands, stimulating the cortisol secretion and preparing the body for a “fight or flight” response to external stimuli. As cortisol levels rise, this change is detected by receptors in the hypothalamus and hippocampus, leading to the downregulation of the stress response via a negative feedback mechanism (Kinlein et al., 2019).

However, recent studies suggest that abnormal activation of the HPA axis may be implicated in the pathogenesis of anxiety disorders. Chronic HPA axis hyperactivity correlates with increased anxiety disorder relapse rates, suggesting its potential as a treatment monitoring biomarker for relapse risk. Furthermore, early-life stress can induce lasting HPA axis alterations that persist into adulthood, significantly affecting mental health outcomes (Juruena et al., 2020). Beyond stress, numerous animal model studies have demonstrated that the gut microbiota can influence HPA axis activation. GF mice demonstrate heightened HPA axis activation following acute stress, along with upregulated hippocampal genes regulating HPA axis activity (Luo et al., 2018), correlating with diminished anxiety-like responses (Clarke et al., 2013). The observed changes in GF animals can be partially normalized by colonization with microbiota from control mice. Furthermore, stress-induced corticosterone levels and anxiety-related behaviors can also be modulated through probiotic interventions (Moya-Pérez et al., 2017). These findings suggest a link between gut microbiota and HPA axis activation, highlighting the potential of bacterial interventions to improve the mental health of individuals with anxiety disorders.

Most researchers acknowledge three potential mechanisms linking the gut microbiota with HPA axis activation. First, dysbiosis of the gut microbiota may contribute to increased cytokine release and the synthesis of bioactive molecules, such as IL-1β, IL-6, and TNF-*α*. The release of lipopolysaccharide (LPS) is also elevated, triggering an intestinal inflammatory response (Simkin, 2019). Studies have shown that low diversity in gut microbial communities is associated with increased rodent inflammation, which may cross the BBB and lead to abnormal HPA axis activation (Bailey et al., 2011). Second, dysbiosis can alter SCFAs levels. The anti-inflammatory properties of SCFAs (Section 2.2) are known to reduce microglial activity and limit local inflammatory processes, which in turn can modulate HPA axis activity (M et al., 2018). Third, another significant communication pathway between the gut microbiota, the CNS, and the HPA axis is direct interactions between the ENS and the VN. The VN plays a crucial role in maintaining the homeostasis of the HPA axis and the sympathoadrenal system (SAS). A study in adult rats suggests that during chronic VNS, rats exhibit a faster return to baseline HPA axis responses (Thrivikraman et al., 2013). Human studies similarly show that transcutaneous auricular VN stimulation downregulates HPA activity levels (Yan et al., 2024). Several cutting-edge studies have demonstrated that transcutaneous vagus nerve stimulation (tVNS) can be an effective strategy to modulate the MGB axis and may intervene in the future in the progression or treatment of anxiety disorders (Yue et al., 2023).

#### 4.2.2 Tryptophan (Trp) metabolism

The gut microbiota modulates brain function by influencing neurotransmitter production, with tryptophan metabolism being a key component. Trp, an essential amino acid in protein-rich foods, is primarily absorbed in the small intestine. It crosses the BBB via the L-type amino acid transporter (LAT1/LAT1) to enter the CNS (Agus et al., 2018). Within the host, tryptophan metabolism proceeds through three main pathways: the kynurenine, serotonin, and indole pathways, generating bioactive metabolites (Figure 2).

Trp is the sole substrate for 5-HT in the serotonin pathway, which occurs in 90% of enterochromaffin cells in the distal gastrointestinal tract and 10% in the CNS. Numerous studies have shown that changes in serotonin levels in humans are associated with the pathogenesis of anxiety disorders. Studies have shown decreased levels of 5-HT in anxious rats’ ventral hippocampus (VH) (Rex et al., 2005), which may be related to dysregulation of the HPA axis. The Bed Nucleus of the Stria Terminalis (BNST), a major subdivision of the extended amygdala, is proposed to regulate responses to anxiogenic environments in humans and rodents. Garcia-Garcia et al. (2018) showed that dBNST 5-HT1A receptors mediate both the anxiolytic effects of 5-HT and 5-HT-modulated anxiety-like behaviors. Changes in the gut microbiota may play a direct role in anxiety-like behaviors and alterations in serotonin. Tao et al. (2024) identified a strong association between the presence of *Jeotgalicoccus* and *Staphylococcus* and serum 5-HT levels within a co-occurrence network. Furthermore, as shown in a human enterochromaffin cell model, SCFAs have been demonstrated to promote TPH1 transcription and serotonin production in the colon (Reigstad et al., 2015). Thus, the gut microbiota, which acts through SCFAs, is also an essential determinant of intestinal 5-HT production and homeostasis.

Kynurenine is the main biologically active product of Trp catabolism, and approximately 90% of Trp degraded in this manner occurs in the liver, with the remainder being actively transported across the BBB by large neutral amino acid carriers for catabolism into quinolinic acid (QUIN) and kynurenic acid (KYNA) (Ye et al., 2024). The rate of tryptophan metabolism along the kynurenine pathway is dependent on the expression of indoleamine-2,3-dioxygenase (IDO1), found in all tissues, and tryptophan-2,3-dioxygenase (TDO), which is localized to the liver (Kennedy et al., 2017). Studies have shown that pro-inflammatory cytokine levels, IFNγ in particular, increase IDO1 activity, and that glucocorticoid levels can induce TDO expression. Compared with control mice, CRS mice showed significant upregulation of IDO 1 levels in the brain and intestine, facilitated the shift of the TPH metabolic pathway to Kyn signaling, and reversed the anxiety-like behavior of CRS mice after administration of the IDO inhibitor 1-methyltryptophan (1-MT). In addition, it was shown that butyrate down-regulated IDO-1 expression and reduced Kyn pathway activation by lowering STAT1 levels and HDAC inhibitor properties (Martin-Gallausiaux et al., 2018). The above findings suggest a key role for the gut microbiota in regulating the metabolic pathway of canine uridine. As far as we know, there is currently a cohort study that recruited 110 subjects, suggesting that gut microbiota dysbiosis and the Kyn pathway activity can serve as potential biomarkers in patients with major depressive disorder (Lin et al., 2023). Unfortunately, as the most common comorbidity of depression, there is still a lack of large-scale cohort studies exploring the existence of an association between dysregulation of the kynurenine pathway and gut microbiota and psychiatric disorders. Whether dysregulation of the kynurenine metabolic pathway has the potential to be a biomarker for patients with anxiety disorders is unknown.

A recent study by Kaur et al. (2019) demonstrated enriched Trp metabolic activity in the five microbial phyla Actinobacteria, Firmicutes, Bacteroidetes, Proteobacteria, and Fusobacteria, and the genera *of Clostridium*, *Burkholderia*, *Streptomyces*, *Pseudomonas*, and *Bacillus*. Comparing plasma extracts from GF mice with samples from conventional animals, Wikoff et al. (2009) found that a bacterially mediated reduction in the production of tryptophan bioactive indole metabolites, such as indole sulfate and the antioxidant indole-3-propionic acid (IPA). IPA administration modulates microbiota composition in the gut, suppresses microbiota dysbiosis (Zhao et al., 2019), and alleviates anxiety and spatial memory deficits in mice (Fang et al., 2022). These findings indicate that the gut microbiota and its metabolites play an important role in regulating anxiety through the MGB axis by modulating the endocrine system.

### 4.3 The influence of gut microbiota and its metabolites on the immune system

**Figure 3:**
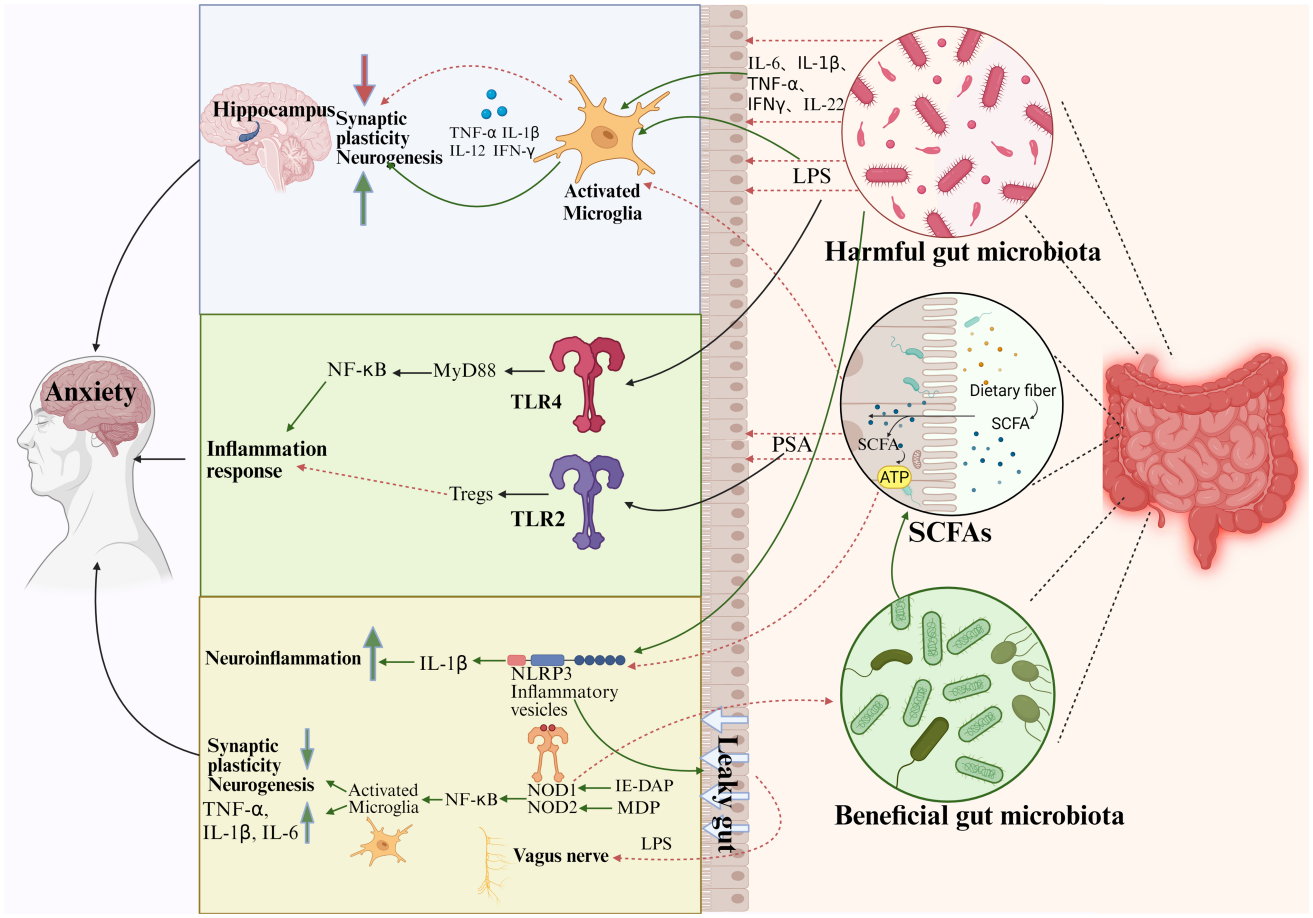
The gut microbiota may modulate the immune system through the following pathways. (1) Harmful gut microbiota can produce inflammatory factors and lipopolysaccharides that promote microglia hyperactivation, modulate hippocampal neurogenesis and synaptic plasticity, and thus affect anxiety. (2) LPS can bind to TLR4 and induce TLR4/MyD88/NF-κB pathway-dependent proinflammatory factor gene expression to promote inflammatory responses in the CNS. PSA can promote inflammation in the CNS via TLR2 signaling and directly induce the anti-inflammatory function of Tregs (3). NLRP3 inflammatory vesicles are key regulators of colonic homeostasis. Gut microbiota induces IL-1β expression via NLRP3 inflammatory vesicles, which promotes “leaky gut” and neurological inflammation. At the same time, endotoxin also inhibits vagal activation, co-regulating anxiety. (4) NOD1 recognizes iE-DAP and NOD2 recognizes MDP, inducing NF-κB pathway-dependent pro-inflammatory factor gene expression, promoting aberrant activation of microglia, and facilitating the expression of inflammatory factors, which affects synaptic plasticity and neurogenesis in the relevant brain regions. (Green lines are facilitative, red lines are inhibitory). LPS, Lipopolysaccharide; PSA, Polysaccharide; SCFAs, short-chain fatty acids; TLR, Toll-like receptors; MyD88, Myeloid Differentiation Factor 88; NOD, nucleotide-binding oligomerization domain; NF-κB, nuclear factor kappa-B; IE-DAP, γ-D-glutamyl-meso-diaminopimelic acid; MDP, muramyl dipeptide; IL-6, Interleukin-6, IL-1β, Interleukin-1β; IL-22, Interleukin-22; TNF-α, tumor necrosis factor-α; IFN-γ, Interferon-γ (Created with BioRender.com).

The human immune system comprises innate and adaptive immunity, which defend against external threats and maintain internal homeostasis. The gastrointestinal tract harbors the body’s highest density of immune cells and communicates with the gut microbiota through direct contact or secreted compounds. Via the MGB axis, it regulates brain function and behavior, potentially influencing various neurological disorders. Growing evidence suggests that gut microbiota and their metabolites may promote anxiety disorders through immune-mediated pathways (Figure 3).

Firstly, microglia are a key component of the innate immune system. Microglia hyperactivation is considered a risk factor for developing central nervous system disorders. Hyperactivation of microglia produces more peripheral inflammatory cytokines such as TNF-*α*, IL-1β, IL-12, and IFN-*γ*, further contributing to impaired neurogenesis in the hippocampus and promoting stress vulnerability (He et al., 2024a). It has been shown that the lack of gut microbiota affects the microglia transcriptome, leading to the interconversion of microglia subpopulations. These alterations can be effectively reversed by gut microbiota colonization (Huang et al., 2023). He et al. (2024b) showed that the hippocampus of stress-sensitive animals displays more extensive microglia activation, abnormal interactions between microglia and neurons, and lower synaptic plasticity. Transplantation of fecal microbiota from stress-resistant mice into naïve mice protects microglia from activation and preserves synaptic plasticity in the hippocampus. *L. reuteri* supplementation reduced anxiety-like behavior and microglial activation while increasing dendritic spine density in mice, paralleled by elevated butyrate levels in the gut, serum, and brain. Butyrate alone replicated these effects, implicating it as a key mediator (Duan et al., 2021).

Second, microglia can be activated by a variety of extracellular stimuli, many of which are mediated by Toll-like receptors (TLRs), which have been identified as pattern recognition receptors (PRRs) for pathogen-associated molecular patterns and mediate inflammatory and immune responses following infection (Rivest, 2009). It was shown that deletion of TLR2 and TLR4 eliminated repeated social defeat stress (R-SDS)-induced social avoidance and anxiety in mice. TLR2/4 is a key mediator of R-SDS-induced activation of microglia in the mPFC, leading to anxiety (Nie et al., 2018). Interestingly, the knockdown of TLR4 in Tph2 neurons, which are highly expressed in the DRN, reduces anxiety-like behavior in male but not female mice, suggesting that TLR4 in Tph2 neurons regulates anxiety-like behavior in a sex-dependent manner (Li et al., 2022). TLR expression has been shown to influence the progression of psychiatric disorders, with studies reporting that the expression of certain TLRs normalizes during pharmacological treatment for major depression (Yy et al., 2016). These findings suggest that TLRs could serve as biomarkers for evaluating the efficacy of novel psychiatric treatments. However, research on their role in anxiety disorders remains limited.

TLRs serve as a critical interface between the intestinal epithelial barrier, the microbiota, and the immune system, playing a pivotal role in shaping the gut microbiota. TLRs can recognize gut microbiota and their metabolites, and the interactions between TLRs and the gut microbiota are essential for maintaining gut immune homeostasis (Rakoff-Nahoum et al., 2004). Kim et al. (2012) demonstrated that a high-fat diet alters the composition of the intestinal microbiota, leading to elevated levels of lipopolysaccharide (LPS) in the lumen of the colon. Gut inflammation was triggered through the TLR4/MyD88/NF-κB signaling pathway, which in turn altered tight junction proteins and enhanced intestinal LPS permeability, ultimately exacerbating the systemic inflammatory response in mice. The symbiosis factor of *Bacteroides fragilis*, polysaccharide A (PSA), can directly induce the anti-inflammatory function of T regulatory cells (Tregs) by signaling through TLR2 on CD4 + T cells (Round et al., 2011). Probiotic consumption during adolescence alters toll-like receptor 4 activity in the paraventricular nucleus (PVN) of the hypothalamus of male mice in response to stressors in adulthood to alleviate anxiety-like behavior (Murray et al., 2019).

Finally, in addition to TLRs, NOD-like receptors (NLRs) play a crucial role in sensing molecules associated with intracellular infections and stress, and they are key regulators of immune function. Gram-positive and Gram-negative bacteria express peptidoglycan, but they produce distinct motifs that can be recognized by either NOD1 or NOD2 (Almeida-da-Silva et al., 2023). The NLRP6 inflammasome is a key regulator of colonic homeostasis. Studies have shown that the gut microbiota is essential for inflammasome activation. In GF mice, the absence of gut microbiota leads to a loss of caspase-1 auto-cleavage and reduced inflammasome activity, resulting in decreased colonic IL-18 levels and dysregulated colonic homeostasis (Jarret et al., 2020). NLRP3is a key regulator in maintaining colonic homeostasis. LPS activates the NLRP3 inflammasome when the intestinal flora is dysbiotic or the intestinal barrier is compromised (Swanson et al., 2019). Upon assembly, this inflammasome cleaves to activate caspase-1, which in turn processes pro-IL-1β and pro-IL-18 into biologically active mature forms (Quan et al., 2025). IL-1β released into tissues is a potent pro-inflammatory cytokine that disrupts the integrity of the intestinal epithelial barrier by down-regulating the expression of tight junction proteins (e.g., occludin and claudin), leading to “leaky gut” (Awad et al., 2023). This allows more bacterial metabolites (e.g., LPS) and inflammatory factors to translocate into the circulatory system and trigger systemic inflammation, thereby promoting anxiety-like behaviors. Overall, the gut microbiota and its metabolites modulate neuroimmune responses through PRRs, influencing glial cell dynamics, cytokine profiles, and synaptic plasticity. The dual role of microglia, which can be either pathogenic or protective, highlights that their function is highly context-dependent, varying with the specific type and duration of immune challenge, and targeting specific PRR pathways may provide novel therapeutic strategies for anxiety disorders.

This chapter elucidates the complex mechanisms of the MGB axis, revealing that the gut microbiota and its metabolites do not act through a single pathway but are regulated through a complex interconnected network of neural, endocrine, and immune communications. The VN acts as a direct information highway, while SCFAs and Trp act as key hormonal and immune messengers. Remarkably, these pathways do not operate independently but are highly interdependent: for example, SCFAs produced in the gut can simultaneously strengthen the BBB, inhibit histone deacetylase, suppress microglia activation, and influence Trp metabolism. This interconnectedness suggests that therapeutic strategies targeting the MGB axis may have synergistic effects, but also increases the difficulty of isolating the contribution of a single mechanism. Disruption of this complex multisystem communication mechanism between the MGB is a central pathological event in anxiety disorders.

## 5 The influence of aerobic exercise on anxiety disorders

Anxiety is a prevalent mental illness that significantly impacts daily functioning and wellbeing. Individuals with anxiety disorders are at an increased risk of cardiovascular disease and premature death (Kandola and Stubbs, 2020). Aerobic exercise, a form of physical activity, has consistently been linked to improved physical health, life satisfaction, cognitive function, and mental health (Carek et al., 2011). Conversely, physical inactivity appears to be related to the development of psychological disorders (Kandola et al., 2019).

Rodent studies show that voluntary wheel running reduces anxiety-like behaviors and enhances cognition. In adult male C57BL/6 mice, 3–4 weeks of aerobic exercise increased open-arm exploration in the elevated plus maze (EPM), while improving center time and locomotion in the OFT compared to sedentary controls (Duman et al., 2008). Long-term aerobic exercise similarly reduced anxiety-like behavior in mice and exerted an anxiolytic effect at different ages (Patki et al., 2014; Zolfaghari et al., 2021). In addition, the beneficial effects of exercise on anxiety-like and cognitive behaviors were also observed in older animals. Evidence of the impact of aerobic exercise on anxiety was found in 18-month-old animals, showing an increase in center time and a decrease in the time spent in the closed arm of the EPM (Pietrelli et al., 2012). However, a contrasting hypothesis suggests that very prolonged, lifelong exercise might, under certain conditions, paradoxically increase anxiety. This has been theorized to be linked to excessive hippocampal neurogenesis or to the cumulative effects of exercise-induced fatigue. Further research is needed to clarify whether exercise-induced neurogenesis is relevant to behavioral tests of anxiety in rodents (Morgan et al., 2018).

Clinical studies in anxiety disorder patients show consistent benefits of aerobic exercise. During COVID-19, a cohort study revealed that aerobic exercise produced greater reductions in anxiety and depression than strength training. Notably, 16 weeks of aerobic exercise achieved anxiolytic effects comparable to pharmacotherapy while significantly improving physical health markers (body weight, blood pressure, and cardiovascular parameters) beyond medication effects (Verhoeven et al., 2023). A 12-week randomized controlled trial revealed that both low- and moderate/high-intensity exercise similarly improved anxiety symptoms at follow-up, with no significant dose–response effect (Henriksson et al., 2022). These findings contrast with Aylett et al. (2018), which concluded that higher-intensity exercise yielded superior anxiolytic effects. The discrepancy could stem from differences in the patient populations, exercise protocols, or outcome measures used across the studies. Collectively, aerobic exercise represents a viable alternative for anxiety disorder patients reluctant to pursue medication/psychotherapy or lacking access to first-line treatments. Although clinical studies confirm its therapeutic benefits, the optimal exercise intensity requires further investigation through more rigorous clinical trials.

Data from both rodent and clinical studies provide strong evidence that aerobic exercise is an effective non-pharmacological intervention for anxiety relief. The benefits span a wide range of ages, and the efficacy is comparable to first-line treatments such as medications, with the added benefit of improving physical health. However, key unanswered questions remain about the optimal dose of exercise. Conflicting findings regarding exercise intensity suggest that the relationship may be nonlinear and subject to individual difference factors. These include fitness levels, genetic susceptibility, and anxiety subtypes in people with anxiety disorders. These research gaps suggest that to maximize the anxiolytic benefits of exercise, individualized exercise prescription programs may need to be further explored. In summary, aerobic exercise is an effective and feasible strategy for the treatment of anxiety disorders. A large body of evidence supports its efficacy, with significant reductions in anxiety-like behaviors and anxiety symptoms in both preclinical and clinical settings. Future research needs to focus on clarifying the optimal aerobic exercise intensity, which can be further validated through more rigorous clinical trials.

## 6 The influence of aerobic exercise on gut microbiota and its metabolites

**Figure 4:**
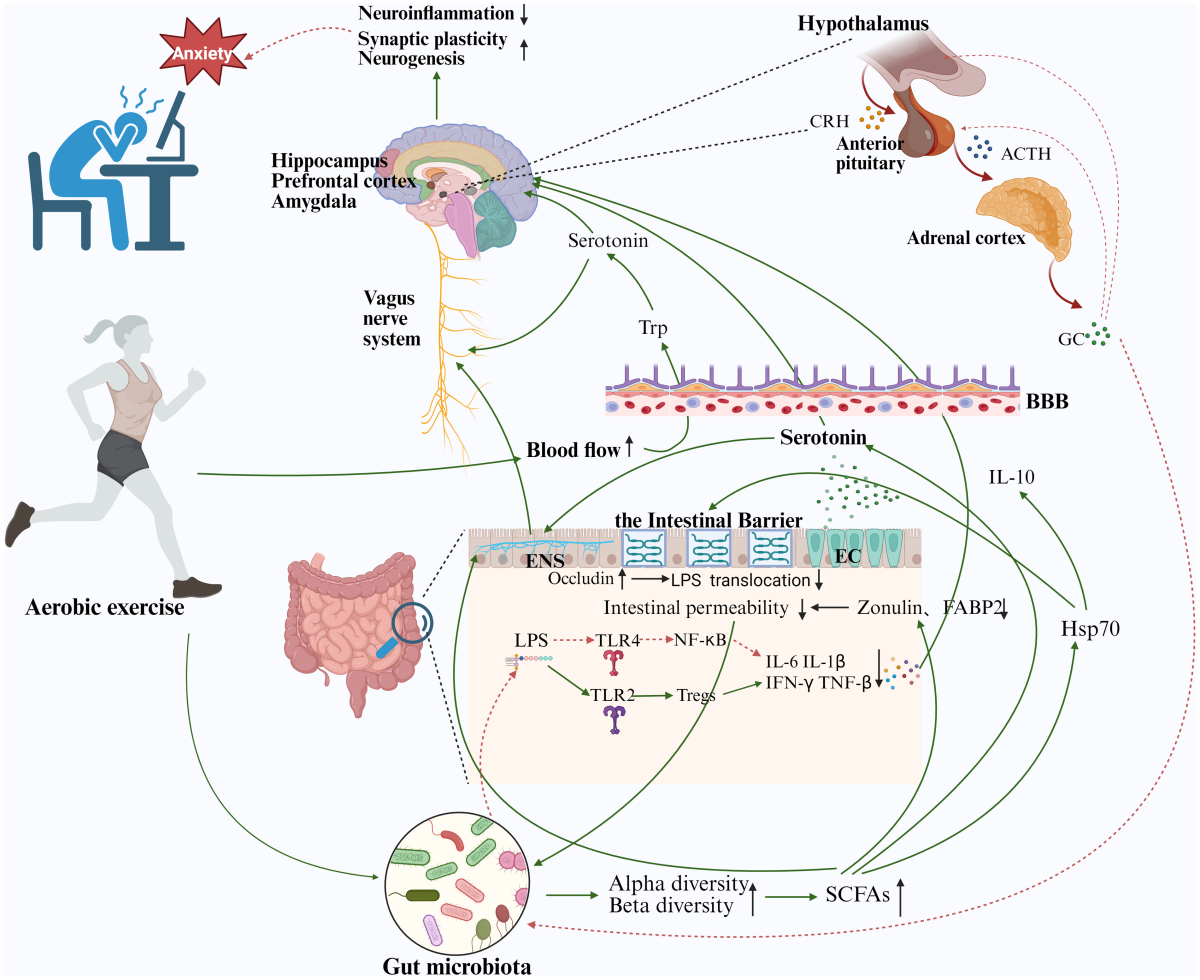
The effects of aerobic exercise on anxiety disorders via the microbiota-gut-brain axis. First, during aerobic exercise, circulatory and cerebral blood flow increase, increasing the availability of tryptophan in various regions of the brain and (1) increasing central pentraxin levels. (2) Increased levels of serotonin activate the VN system, thereby modulating neuroinflammation, synaptic plasticity, and neurogenesis. Second, aerobic exercise increases the diversity of the gut microbiota, regulates the balance between beneficial and pathogenic bacteria, and increases the level of SCFAs, thereby (1) promoting the synthesis and secretion of intestinal serotonin. (2) Enhance intestinal barrier function by stimulating intestinal epithelial cells to synthesize heat shock protein and the secretion of IL-10. (3) Enhances intestinal barrier function by decreasing zonulin and FABP2, plasma biomarkers of intestinal barrier permeability. Third, aerobic exercise can increase the diversity of gut microbiota, regulate the balance between beneficial and pathogenic bacteria, reduce the production of lipopolysaccharides, inhibit the activation of the NF-κB pathway through TLR4 and the activation of Tregs through TLR2 to collectively inhibit the secretion of inflammatory factors IL-6, IL-1β, TNF-α, and IFN-γ. The reduction of inflammatory factors inhibits the activation of the HPA axis and suppresses the production of cortisol, thereby modulating anxiety. Decreased cortisol levels are also able to promote intestinal homeostasis, resulting in bidirectional gut-brain communication (green lines are facilitative, red lines are inhibitory). BBB, Blood–Brain Barrier; ENS, Enteric nervous system; EC, Enterochromaffin cell; LPS, Lipopolysaccharide; NF-κB, Nuclear factor kappa-B; CRH, Corticotropin-releasing hormone; ACTH, Adrenocorticotropic hormone; GC, Glucocorticoid; Trp, Tryptophan; Tregs, Regulatory cells; Hsp70, Heatshock protein 70 (created with BioRender.com).

Accumulating evidence suggests that host-microbe symbiosis significantly impacts mental health, with the microbiota-gut-brain axis serving as a critical bidirectional pathway linking the gastrointestinal microbiota to brain function and homeostasis (Figure 4). The gastrointestinal microbiota not only influences human behavior but may also contribute to the pathophysiology of mental disorders (Malan-Muller et al., 2018). Recent advancements in microbiome research have expanded the concept of the gut-brain axis to the “microbiota-gut-brain axis,” highlighting the critical role of the microbiome in regulating gut-brain communication. Additionally, microbiota diversity was significantly higher in athletes compared to individuals with high and low BMI. Notably, the genus *Akkermansia* was more prevalent in elite athletes, and levels of SCFAs such as acetate, propionate, butyrate, and valerate were significantly elevated in athletes compared to controls (Barton et al., 2018). These findings suggest that exercise may influence gut microbiota composition and metabolite levels.

The positive effects of aerobic exercise on gut health primarily involve enhancing colon function, increasing microbiota diversity, and regulating the balance between beneficial and pathogenic microbes. In preclinical studies, aerobic exercise improved alpha diversity and the Bacteroidetes/Firmicutes ratio in the distal gut and fecal microbiota of high-fat diet-induced obese mice (Denou et al., 2016). Regarding gut microbiota composition, aerobic exercise significantly increased the relative abundance of butyrate-producing bacteria, such as Bacteroidales S24-7 (Evans et al., 2014). Consistent results were also obtained in the non-obese model (Matsumoto et al., 2008). These changes in the gut microbial environment may contribute to the beneficial effects of aerobic exercise on both gastrointestinal and mood disorders. The gut microbiome has been shown to modulate mental health and influence the pathophysiology of anxiety disorders. Interestingly, researchers observed the sex-specific effects of aerobic exercise on mice’s anxiety-like behavior and gut microbiota. Aerobic exercise reduced anxiety-like behavior and altered grooming in males and reduced fear of the open arm in the EPM test in male mice. Furthermore, Prevotellaceae_NK3B31_group and Rhizobiaceae exhibited sex-dependent differences, with females having lower levels of Prevotellaceae NK3B31 group and Rhizobiaceae but higher levels of UCG-005 compared to males (Williams et al., 2023). A key issue is that, although depression and anxiety are more prevalent in females, most interventions aimed at reducing symptom severity and preventing or recovering from these mental health conditions have been developed using male animal models, which limits their applicability to females. Similar findings have been observed in clinical studies. Morita et al. (2019) demonstrated that aerobic exercise increased *Bacteroides* levels in older, healthy women, and this increase was associated with greater distance achieved during the 6-Minute Walk Test (6MWT). Alpha diversity was positively correlated with VO2 peak, and the *Roseburia*, *Sutterella*, and *Odoribacter* genera were also linked to VO2 peak, suggesting that changes in gut microbiota composition are associated with cardiorespiratory endurance (Resende et al., 2021). While the beneficial effects of aerobic exercise and antidepressants on anxiety disorders were comparable, aerobic exercise demonstrated additional, superior benefits for physical health. Specifically, the aerobic exercise group showed improvements in metrics like body weight and cardiovascular parameters, whereas the antidepressant group showed deterioration in these areas (Verhoeven et al., 2023).

Regarding the effect of duration of aerobic exercise on the composition of the gut microbiota, Cataldi et al. (2022) showed that the relative abundance of *Prevotella* appeared to be related to the duration of training, with marathon runners showing increased levels of *Prevotella* and increased levels of diversity (Kulecka et al., 2020). As for exercise intensity, no significant changes in gut microbiota abundance or diversity were observed when exercise was performed at the lowest dose recommended by the World Health Organization (Cataldi et al., 2022). However, moderate-intensity aerobic exercise was found to modify gut microbiota composition and activate the AMPK/CDX2 signaling pathway, enhancing the intestinal barrier (Wang et al., 2022b). These findings highlight a correlation between anxiety and microbial diversity. These changes in gut barrier function compromise epithelial cell integrity, leading to low-grade systemic inflammation. Plasma biomarkers of intestinal barrier permeability, such as zonulin and FABP2, are elevated in patients with anxiety disorders, impaired intestinal barrier function, and altered lipopolysaccharide and gut microbiota composition (Stevens et al., 2017). These changes in gut barrier function compromise epithelial cell integrity, leading to low-grade systemic inflammation. Increased levels of pro-inflammatory cytokines in both the periphery and the brain contribute to the development of anxiety disorders. Aerobic exercise has been shown to increase the abundance of butyrate-producing fecal microbiota and raise cecal butyrate levels, including species such as *Enterobacter*, *Bacteroides*, *Roseburia*, *Prevotella*, *Paraprevotella*, and *Akkermansia* (Matsumoto et al., 2008; Yu et al., 2019). Notably, although Enterobacter are often regarded as conditional pathogens associated with inflammation, their increased abundance may reflect a specific complex microbial community shift induced by exercise, which ultimately promotes elevated butyrate production and creates a favorable metabolic environment. This highlights the context-dependent nature of microbial ecology. Butyrate production in the colon is associated with gut barrier health and the synthesis of heat shock protein 70 (Hsp70), which supports the functional and structural properties of intestinal epithelial cells and promotes the production of IL-10, a key intracellular anti-inflammatory mediator in response to infection, ischemia, and other environmental stresses (Wang et al., 2018). Butyrate stimulates Hsp70 production in epithelial cells, providing structural and functional stability to intestinal epithelial cells under stress (Adesina et al., 2023). Aerobic exercise increases circulation throughout the body and increases Trp availability in various brain regions, leading to elevated levels of 5-HT in the hippocampus and brainstem. This is consistent with research conducted in younger men, which has shown that aerobic exercise leads to a significant increase in Trp availability in the brains of older men (Melancon et al., 2012). These findings highlight the impact of aerobic exercise on gut microbiota composition and its potential role in the pathogenesis of anxiety disorders. However, current evidence on the mechanisms by which aerobic exercise improves gut microbiota in anxiety disorders remains insufficient and warrants further investigation.

The findings synthesized in this chapter firmly establish aerobic exercise as a potent non-pharmacological modulator of the gut microbiota, with direct implications for the pathophysiology of anxiety disorders. The most consistent observations—increased microbial diversity, a shifted Bacteroidetes/Firmicutes ratio, and the enrichment of SCFA-producing genera like Akkermansia and Roseburia—collectively point towards a mechanism through which exercise counteracts the dysbiotic profile often associated with anxiety. The subsequent elevation in SCFA levels, particularly butyrate, provides a plausible causal link, as these metabolites are known to enhance intestinal barrier function, suppress neuroinflammation, and regulate the HPA axis—all key pathways detailed in our earlier chapters. However, the current evidence base primarily reveals correlation. The critical step for the field is to transition from observing these associations to demonstrating a direct, causative role of the gut microbiota in mediating exercise’s anxiolytic effects. Future research employing sophisticated study designs, such as fecal microbiota transplantation from exercised donors into germ-free or antibiotic-treated animal models, is essential to confirm that the anxiety-relieving benefits of exercise can be transferred via the gut microbiota. In summary, aerobic exercise consistently promotes metabolic homeostasis in the gut microbial ecosystem, thereby benefiting mental health. Rather than being an incidental effect of exercise, the modulation of gut microbiota is a central mechanism that translates physical activity into anxiety relief via the flora-gut-brain axis. To advance this promising area of research, future studies need to focus on establishing causality, resolving the functional outputs of microbes, and personalizing exercise interventions based on individual host factors such as gender, diet, and baseline microbiome.

## 7 Exerkines as systemic integrators of the exercise and gut microbiota and anxiety

The specific changes in gut microbiota composition and metabolites induced by aerobic exercise were detailed previously. However, these changes do not occur in isolation, but are embedded in a broader physiological response of the exercise system - a response that is primarily mediated by exerkines. Exerkines are a class of biologically active molecules that are released by tissues such as skeletal muscle and adipose in response to physical exercise (Vints et al., 2022). Therefore, a comprehensive understanding of aerobic exercise modulation of the MGB axis affecting anxiety requires the integration of these microbiota changes with the parallel effects of exercise factors. These exerkines function as powerful endocrine mediators, orchestrating a parallel and interconnected anxiolytic strategy.

Irisin is a myokine that has received much attention in recent years. Exercise has the capability of upregulating proliferator-activated receptor gamma coactivator-1*α* (PGC-1α), leading to increased expression of the downstream protein fibronectin type III domain-containing 5 (FNDC5), which can be cleaved and secreted from the muscle into the bloodstream in the form of the myokine irisin (Pignataro et al., 2021). Uysal et al. (2018) showed that regular aerobic exercise was associated with reduced anxiety and elevated levels of irisin in the brain and white adipose tissue. Aerobic exercise upregulates Irisin via the PGC-1α/FNDC5 pathway, not only penetrates the BBB to promote neuroprotective BDNF expression in the hippocampus but also strengthens the intestinal epithelial barrier (Siteneski et al., 2020; Wrann et al., 2013). Irisin modulates the composition of the gut microbiota. Studies have shown that the relative abundance of *Lactobacillaceae*, *o-red Helicobacter*, and *Staphylococcaceae* is increased in a mouse model of colitis, and irisin treatment reversed the altered gut microbiota (Huangfu et al., 2021). Liu et al. (2023) showed that irisin maintains intestinal barrier integrity and inhibits the production of pro-inflammatory cytokines IL-1β, IL-6, and TNF-α. This enhanced intestinal integrity complements the SCFAs-driven barrier improvement described previously, and together they reduce systemic inflammation and create a healthier microbial environment, thereby alleviating anxiety symptoms. FNDC5/irisin knockout mice have dysbiosis of the gut microbiota, with a decrease in the Firmicutes and an increase in the Bacteroidetes, and exhibit anxiety and depression-like behaviors. The evidence described above suggests a relationship between muscle production of irisin during exercise and the modulation of gut microbiota and anxiety disorders.

IL-6 plays a complex and seemingly contradictory dual role in the relationship between anxiety disorders and gut microbiota. In chronic states of psychological stress, persistently elevated IL-6 acts as a major pro-inflammatory cytokine capable of disrupting intestinal barrier integrity, triggering systemic low-grade inflammation, further disrupting the BBB, and activating microglial cells in the brain, thereby directly contributing to the onset of anxiety (Zhang et al., 2023a). Interestingly, during aerobic exercise, a large circulating increase of up to 100-fold in IL-6, released by contracting skeletal muscle, exhibits strong anti-inflammatory properties, both stimulating the release of anti-inflammatory cytokines (e.g., IL-10) and inhibiting the production of TNF-α (Pedersen and Febbraio, 2008; Severinsen and Pedersen, 2020). Elevated levels of TNF-α in IL-6-deficient knockout mice (Mizuhara et al., 1994). Serum IL-6 concentrations are elevated after exercise, and IL-6 subsequently reaches the intestinal tract, leading to increased levels of Glucagon-like peptide-1(GLP-1) through stimulation of intestinal L-cells, which contribute to the relief of anxiety disorders and memory disorders (Do et al., 2025; Ellingsgaard et al., 2011; Liu et al., 2024). In summary, the exercise-induced acute IL-6 spike helps to create a systemic anti-inflammatory environment that not only directly benefits brain health, but also suppresses gut inflammation, thereby shaping a more beneficial, SCFAs-rich producing gut microbiome that indirectly alleviates anxiety symptoms.

In conclusion, exercise hormones, represented by the muscle-derived cytokines Irisin and IL-6, act as key endocrine messengers, integrating the systemic effects of aerobic exercise with the regulation of gut microbiota and the anxiolytic effects in a synergistic way, transforming the physical stimulation of exercise into a multidimensional defense mechanism against anxiety disorders.

## 8 Conclusion and future perspectives

This review provides a comprehensive summary of the potential mechanisms by which aerobic exercise influences gut microbiota and its metabolites in patients with anxiety disorders, based on evidence from both rodent and human studies. Aerobic exercise modulates the composition of the gut microbiota and its metabolite levels, which may influence anxiety by affecting the gut-brain axis, a complex interplay among the nervous, immune, and endocrine systems. Aerobic exercise increased the diversity of gut microbiota, increased the relative abundance of butyrate- and acetate-producing flora, and elevated the levels of SCFAs, which in turn enhanced intestinal barrier function and suppressed neuroinflammation; and improved hippocampus through a multiplex interaction of vagal, immune (e.g., AMPK/CDX2 signaling pathway, TLR4/NF-κB pathway) and endocrine pathways (HPA axis, tryptophan metabolism) neuroplasticity, and microglia homeostasis.

Current evidence supports the use of aerobic exercise to alleviate symptoms of stress and anxiety. Aerobic exercise can be an effective alternative therapy for people with anxiety disorders who are hesitant to start medication or psychotherapy or for those in areas where first-line resources are not available.

Looking ahead, research should prioritize establishing direct causal links within the ‘exercise-gut microbiota-anxiety’ axis. This can be achieved through sophisticated experimental designs, such as the use of sterile animals, the use of antibiotic-induced colony depletion models, and fecal microbiota transplantation studies, which provide convincing evidence.

Besides, there is still much controversy regarding the optimal intensity and duration of aerobic exercise. Future research should aim to determine the specific intensity and duration required to have a favorable effect on the microbiota and its metabolites and thus reduce anxiety symptoms. Determining the microbiota-gut-brain axis mechanisms by which aerobic exercise modulates anxiety will also inform the development of personalized therapies such as the administration of probiotics or prebiotics. We recommend that future studies standardize genome sequencing methods to obtain microbiota markers and reduce study heterogeneity.

In addition, to bridge the gap between preclinical studies and clinical applications, it is critical to emphasize longitudinal studies and randomized controlled trials in populations. Studies with multi-omics technologies (e.g., macro-genomics, metabolomics, proteomics) should be integrated to systematically reveal the complete network of mechanisms by which aerobic exercise modulates the MGB axis and to elucidate the precise molecular mechanisms by which aerobic exercise modulates the MGB axis to alleviate anxiety disorders.

However, residual confounders such as ethnic differences and various environmental exposures (e.g., dietary habits, physical activity, past and current drug use) should be carefully controlled for in gut microbiota studies. The diversity of the gut microbiota also changes with age, so some of the inconsistencies in the results of previous studies may be due to age differences in the study samples. In the future, larger sample sizes and more rigorous experimental designs are needed to explore the effects of exercise intensity and duration on the gut microbiota and anxiety symptoms and to elucidate the molecular mechanisms by which aerobic exercise alleviates anxiety disorders. This will lead to new strategies for the clinical management of anxiety disorders, which will ultimately benefit patients.
